# On the Administration of the Salicylates in Acute Rheumatism*Extracts from a paper read before the Cambridge Medical Society, by P. W. Latham, M. A., M. D.

**Published:** 1895-10

**Authors:** 


### ON THE ADMINISTRATION OF THE SALICYLATES IN ACUTE RHEUMATISM.*

We have now become so familiar with the successful treatment of acute rheumatism, by means of salicylic acid and salicylates, that it may seem somewhat superfluous for me to address you on the subject. But cases have come under my observation in which objections have been taken to the use of these remedies, on the ground either that they disagreed with the patient, producing nausea, vomiting, etc., or that notwithstanding fairly large doses of the drug, the pains have not been relieved, the temperature has not been reduced, or, most serious of all, cardiac or other complications have arisen during the time the patient was taking the remedy, and when, apparently, he was under its influence. Now, it is in preventing the development of these complications that, when properly administered, the remedy so strikingly shows its power, truly acting as a distinct specific.

In my Croonian Lectures, in 1886, I spoke, apropos of rheumatism, as follows:

"Here is a disorder which, under different treatment, may exist for weeks, stationary, so to speak, in its intensity, the great heat, and nervous and vascular excitement, and pain and swelling exactly of the same amount to-day as they were weeks ago; a disorder which, less than fifty years ago, was said to be often such in itself, and such in its appalling incidents, as to need, from time to time, that medicine should put forth the full compass of all its powers. Every organ, or system of organs, which either directly or indirectly can receive the impression of remedies, are from time to time called to bear all that they can possibly endure, and it is often only when the powers of medicine are pressed even to the verge of destroying life that life is saved.

"And now, with or without the administration of a purgative, as the occasion requires, the patient is placed fully under the influence of salicylic acid, and in from forty to sixty hours, not unfrequently in a shorter time, the pains in the joints have

*Extracts from a paper read before the Cambridge Medical Society, by P. W. Latham, M. A., M. D.

subsided; the limbs can be freely moved, and the bodily temperature has reached the normal condition. But more than this—and here the remedy shows its signal power—in no case of rheumatism that has come under my care during the last six years, either in hospital or in private practice, has there been developed, where the heart was previously sound, any cardiac complication, such as endocarditis or pericarditis. If this can be maintained and ensured, we have indeed, in our hands, a most potent remedy. Cardiac complications constitute the chief danger of acute rheumatism, and the danger, if the disease is taken in hand soon enough, may, with our new remedy, be averted."

Eight years further experience has only confirmed what was then stated. I have seen numbers of cases where complications have been developed before the patients came under my care, but I feel strongly that these complications might be prevented, or at least materially lessened, by earlier and more energetic treatment, and it is for this reason chiefly that I venture to address you to-day.

Now what are the conditions to insure success?

Principally, the true salicylic acid obtained from the vegetable kingdom must alone be employed. If you have to give large doses, avoid giving the artificial product obtained from carbolic acid, however much it may have been dialysed and purified. An impure acid will very quickly produce symptoms closely resembling delirium tremens.

"The causes of failure with this remedy, so far as I have been able to judge, are:

1st. Insufficient doses at the commencement.

2d. The non-administration of a purgative.

3d. Feeding with substances other than milk, such as beef-tea, broths, etc., especially in earlier stages.

As this plan of treatment works prosperously day after day in its immediate effects, so day after day it gives an earnest of the remedial impression it is exercising upon the whole disease. It abates the fever, it softens the pulse, it reduces the swelling, and it lessens the pain. In short, it subdues the vascular system like a bleeding, and pacifies the nervous system like an opiate; and often in the course of a week, the acute rheumatism is gone. In three days there is often a signal mitigation of all the symptoms; and in a week I have often seen patients, who have been carried helpless into the hospital and shrinking at the least jar or touch, or movement of their limbs, risen from their beds, and walking about the wards quite free from pain.

Now, if in the treatment of acute rheumatism, you were to choose one indication and abide by it, and were to trust one class of remedies and to it only, you would find more cases that admit of a readier cure by the method now described than by either of the two former. You would find the aggregate of morbid actions and sufferings, which constitute the disease, more surely reached and counteracted, and more quickly abolished by medicines operating upon the abdominal viscera only, than by those which influence either the blood vessels only, or the nerves only.

I would still recommend that the natural salicylic acid, or its salt, should be employed, in preference to the artificial acid, when large doses are administered. I admit that what are termed the "physiologically pure" may be as good, but I prefer using the natural products, owing to the complete safety which, with ordinary care, attends their administration. In a paper in the *British Medical Journal* of December 10th, 1881, I first called attention to the danger of using the artificial acid. The impurities then existing in it amounted to as much as 15 per cent. By improved methods of preparing it, in 1884, these impurities were reduced to 5 per cent, and now it is so carefully prepared, that the product is said to be "physiologically pure." In the *Pharmaceutical Journal* of November 22d, 1890, you will find a very exhaustive paper by Professor Dunstan, giving an account of these impurities, with a report, also, by Professor Charteris, of the poisonous effects which two of these impurities, viz., orthocreosotic acid and paracreosotic acid, have on the animal system. The same journal also contains a report of an interesting discussion on the subject which took place at the Pharmaceutical Society.

